# Management of Preeclampsia, Severe Preeclampsia, and Eclampsia at Primary Care Facilities in Bangladesh

**DOI:** 10.9745/GHSP-D-19-00124

**Published:** 2019-09-23

**Authors:** Anna Williams, Marufa Aziz Khan, Mohammed Moniruzzaman, Sk Towhidur Rahaman, Imteaz Ibne Mannan, Joseph de Graft-Johnson, Iftekhar Rashid, Barbara Rawlins

**Affiliations:** a Save the Children, Dhaka, Bangladesh.; b Pathfinder, Dhaka, Bangladesh.; c Jhpiego, Kabul, Afghanistan.; d Save the Children, Washington, DC, USA.; e United States Agency for International Development/Bangladesh, Dhaka, Bangladesh.; f Jhpiego, Washington, DC, USA.

## Abstract

**Introduction:**

Eclampsia-related conditions are the second leading direct cause of obstetric deaths in Bangladesh. Efforts to prevent such deaths in low- and middle-income countries are increasingly focused on task shifting at the primary care level to enable frontline providers to screen and initiate treatment for women with preeclampsia, severe preeclampsia, and eclampsia (PE/SPE/E). The MaMoni Health Systems Strengthening project (funded by the United States Agency for International Development) implemented a magnesium sulfate intervention at primary care facilities in 4 Bangladesh districts in 2016 and 2017.

**Methods:**

The project trained frontline providers through a cascade approach from the national to the union level. A PE/SPE/E patient algorithm, digital blood pressure machines, and eclampsia kits with magnesium sulfate were supplied to service providers at each facility. We conducted a retrospective record review of facility-level data to assess the degree to which newly trained frontline providers adhered to a protocol that incorporated the use of magnesium sulfate for SPE/E in primary care settings.

**Results:**

In total, 283 women were found to have PE/SPE/E. Fifty-four percent were managed according to the protocol. The required supplies were present at each facility, but some issues existed with regard to availability and functionality of blood pressure apparatuses.

**Discussion:**

Challenges related to recordkeeping and service quality limited the analysis. Frontline providers need refresher trainings, ongoing supervision, properly calibrated blood pressure devices, and performance monitoring support in order to improve screening and management of PE/SPE/E in primary care facilities.

## Abstract (precis)

Program introduction, including cascade training, to screen for severe preeclampsia and eclampsia and initiate treatment with magnesium sulfate was somewhat successful. Challenges included inconsistent adherence to the national protocol, data quality, and some issues with supplies and equipment.

## INTRODUCTION

Preeclampsia, severe preeclampsia, and eclampsia (PE/SPE/E) are hypertensive disorders of pregnancy that contribute significantly to global maternal and perinatal mortality.^1^ Marked by high blood pressure (BP) and the presence of albumin in urine, preeclampsia is a risk factor for the potential development of severe preeclampsia or full-blown eclampsia and should be monitored. Management of SPE/E poses a challenge in low- and middle-income countries due to a lack of basic supplies, health worker shortages, limited competencies of frontline providers, and systems challenges that lead to delays in women receiving necessary treatment.^2^^–^^4^ In Bangladesh, eclampsia-related conditions are the second leading direct cause of obstetric deaths and lead to 24% of all maternal deaths.^5^ Over 1,000 women die each year in Bangladesh due to PE/SPE/E. As in many low- and middle-income countries, most pregnant women who develop PE/SPE/E in Bangladesh do not get diagnosed or treated. They either do not access the health system at all, are not screened properly, or do not receive timely treatment due to delays in (1) making the decision to seek care, (2) being transported to receive care, and (3) actually receiving the required treatment at the care site where it is available.^6^^,^^7^

### PE/SPE/E Detection and Management at the Primary Care Level: A Global Priority

In recent years, global efforts to reduce eclampsia-related deaths have focused on task shifting, or enabling frontline health workers to identify women with PE/SPE/E and initiate management of the disorder.^8^^,^^9^ Calcium supplementation is recommended for preventing preeclampsia when dietary intake of calcium is low, while antihypertensive drugs may be necessary for women with PE. Magnesium sulfate (MgSO_4_) is recommended by the World Health Organization (WHO) to manage SPE/E among pregnant women. In settings where administering a full MgSO_4_ regimen (which includes a “loading dose” followed by scheduled maintenance doses) is not possible, WHO recommendations include providing the initial MgSO_4_ loading dose (via intramuscular injection and/or intravenous drip) and immediately transferring the individual to a higher level of care.^10^ To implement this strategy, frontline health workers in low- and middle-income countries need to have access to BP gauges, urine dipsticks, and MgSO_4_, and need to be trained to screen all pregnant women >20 weeks of gestation for elevated BP, urine albumin, and the presence of any danger signs. If SPE/E is identified, the workers need to administer a MgSO_4_ loading dose and facilitate a timely referral of the woman to a higher-level health facility.

Although the inputs are standard, numerous obstacles may be encountered when rolling this service out in low- and middle-income countries. Weak health systems may have inadequate service delivery protocols, provider skills, systems for supportive supervision, availability of essential supplies (such as BP gauges, urine dipsticks, and injectable MgSO_4_), and collection and use of monitoring data. Barriers to ensuring women with PE/SPE/E are identified and optimally managed likely vary from setting to setting, yet they are expected and must be addressed to further reduce global maternal mortality.

The research group that developed the Preeclampsia Integrated Estimate of Risk (fullPIERS) model for high-income, tertiary care settings also developed the miniPIERS model for providers to use in primary care settings. The miniPIERS is a validated model for identifying women at increased risk of adverse maternal outcomes associated with hypertensive disorders of pregnancy. It relies on a simple assessment of maternal demographics (maternal age, parity, and gestational age), signs (BP and proteinuria), and symptoms (headache, visual disturbance, chest pain, difficulty breathing, upper abdominal pain, nausea, vomiting, and vaginal bleeding with abdominal pain). The miniPIERS study found that using the model in resource-limited settings has the potential to significantly improve care where minimal or no monitoring of hypertensive disorders of pregnancy currently exists.^11^ It has been further tested in community settings through the Community Level Interventions for Preeclampsia clinical trials in India, Pakistan, Mozambique, and Nigeria (https://clinicaltrials.gov/ct2/show/NCT01911494). The results are forthcoming and are expected to make a valuable contribution to the evidence base on effective intervention strategies for identifying and managing PE/SPE/E at the community level in low-resource settings.

Currently, though, evidence is lacking on the effectiveness of program interventions focused on diagnosis, management, and referral of women with eclampsia-related conditions at primary-level health facilities and in communities. This topic is of particular interest in Bangladesh following changes within the past few years in the national PE/SPE/E protocol and recent program efforts. This article examines service delivery data from 35 primary care facilities that received support for providing screening and pre-referral treatment with MgSO_4_ as part of their standard maternal health services. The facilities were supported by the MaMoni Health Systems Strengthening project (MaMoni HSS) to improve the quality and reach of their maternal and newborn health services by using a range of evidence-based interventions, including the introduction of PE/SPE/E screening and management following national guidelines.

Evidence is lacking on the effectiveness of program interventions for the diagnosis, management, and referral of women with eclampsia-related conditions.

## PROJECT DESCRIPTION

**FIGURE 1:**
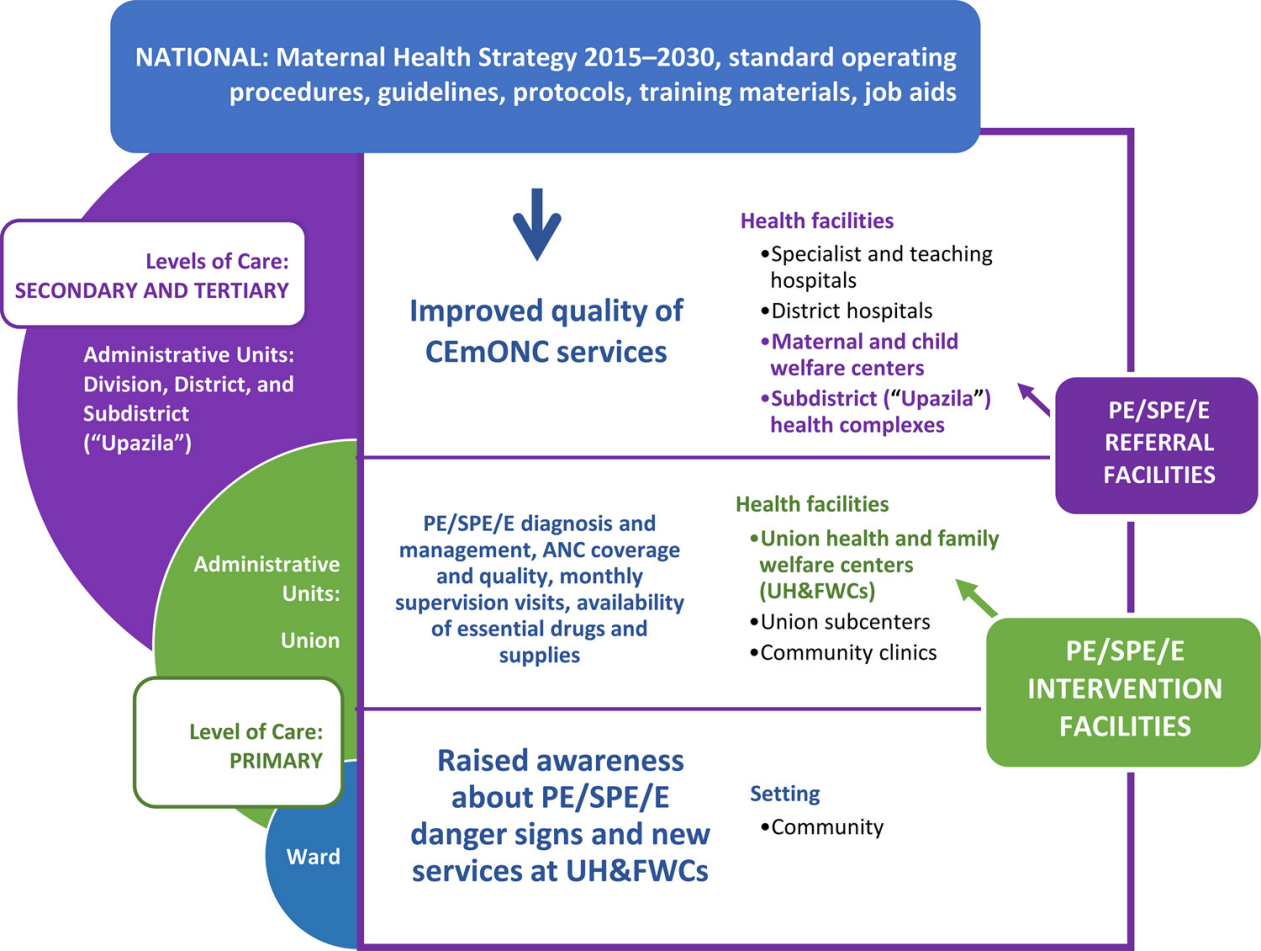
MaMoni HSS Project's Maternal Health Interventions in Bangladesh Abbreviations: ANC, antenatal care; CEmONC, comprehensive emergency obstetric and neonatal care; HSS, Health Systems Strengthening; PE/SPE/E, preeclampsia, severe preeclampsia, or eclampsia.

MaMoni HSS was a large maternal, newborn, and child health (MNCH) project in Bangladesh that was funded by the United States Agency for International Development (USAID) between 2013 and 2018. Its maternal health interventions focused on strengthening public-sector services from the community level to secondary-level referral facilities to provide quality antenatal care (ANC) during labor and delivery, newborn care, and postnatal care (PNC) including postpartum family planning (Figure 1). The project also worked at the national level to support the Ministry of Health and Family Welfare (MOHFW) to develop a maternal health strategy and standard operating procedures (SOPs). The SOPs incorporated a complete package of evidence-based practices and interventions adopted by MOHFW for implementation through the public sector service delivery system at various levels of care. The project's other work at the national level included the development of various guidelines, protocols, training materials, and job aids for the roll-out of the interventions across the country.

### Bangladesh Health System

Although used in Bangladesh since 1998, MgSO_4_ was not available for treating SPE/E at the primary care level via antenatal services prior to 2016 because of a lack of a standard protocol and uncertainties about the skill level and competence of primary care providers. A basic overview of the health system in Bangladesh is necessary background to understand the intervention we discuss here. The secondary and tertiary levels of the health system comprise subdistrict (locally referred to as *Upazila*) health complexes, maternal and child welfare centers, district hospitals, and various teaching and specialist hospitals. At the primary care level are union health and family welfare centers (UH&FWCs), union sub-centers, and community clinics. UH&FWCs (which are the focus of this article) are typically staffed by 1 subassistant community medical officer (SACMO), 1 to 2 family welfare visitors (FWVs), and 1 or more support staff. SACMOs have participated in a 3-year medical training course following secondary school and provide basic primary health care services. FWVs receive 18 months of training after completing secondary school and provide MNCH care services, including family planning, delivery, and immunization. They are the lead providers of ANC services at UH&FWCs. A medical officer, who is a doctor with at least 5 years of professional medical education, serves at some, but not all UH&FWCs (Figure 1).^12^ Across the public health system, the health care workforce has been described as being in crisis due to a shortage of trained providers, including FWVs; an inappropriate skill mix; and inequitable distribution.^13^ FWVs are critical frontline providers staffing nearly 5,000 UH&FWCs around the country.^14^ While they are recognized and counted within national health surveys as medically trained providers, serious gaps in their provision of maternal health services have also been documented.^15^^,^^16^

### PE/SPE/E Case Detection and Management by Frontline Providers in Bangladesh

**FIGURE 2:**
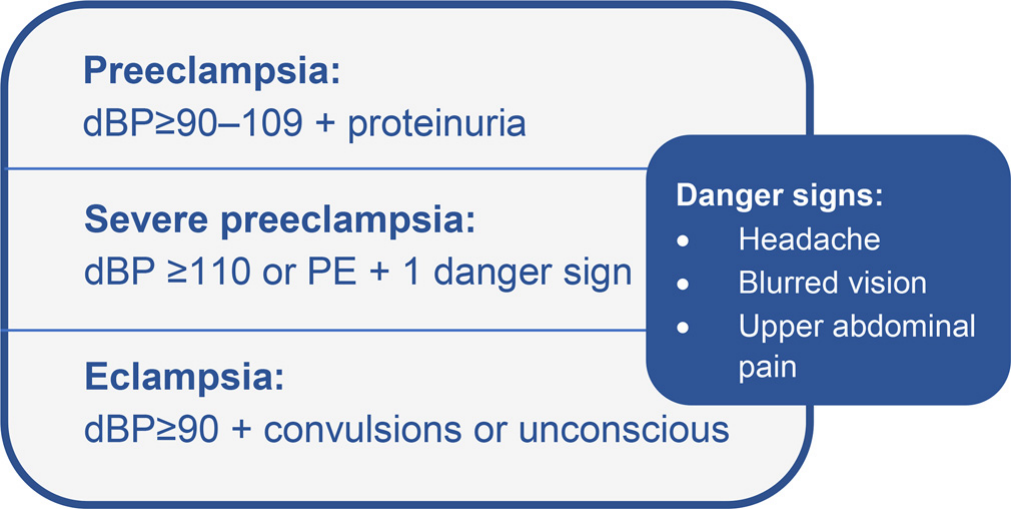
Case Definitions of Preeclampsia, Severe Preeclampsia, and Eclampsia According to National Protocol, Bangladesh Abbreviation: dBP, diastolic blood pressure.

**FIGURE 3:**
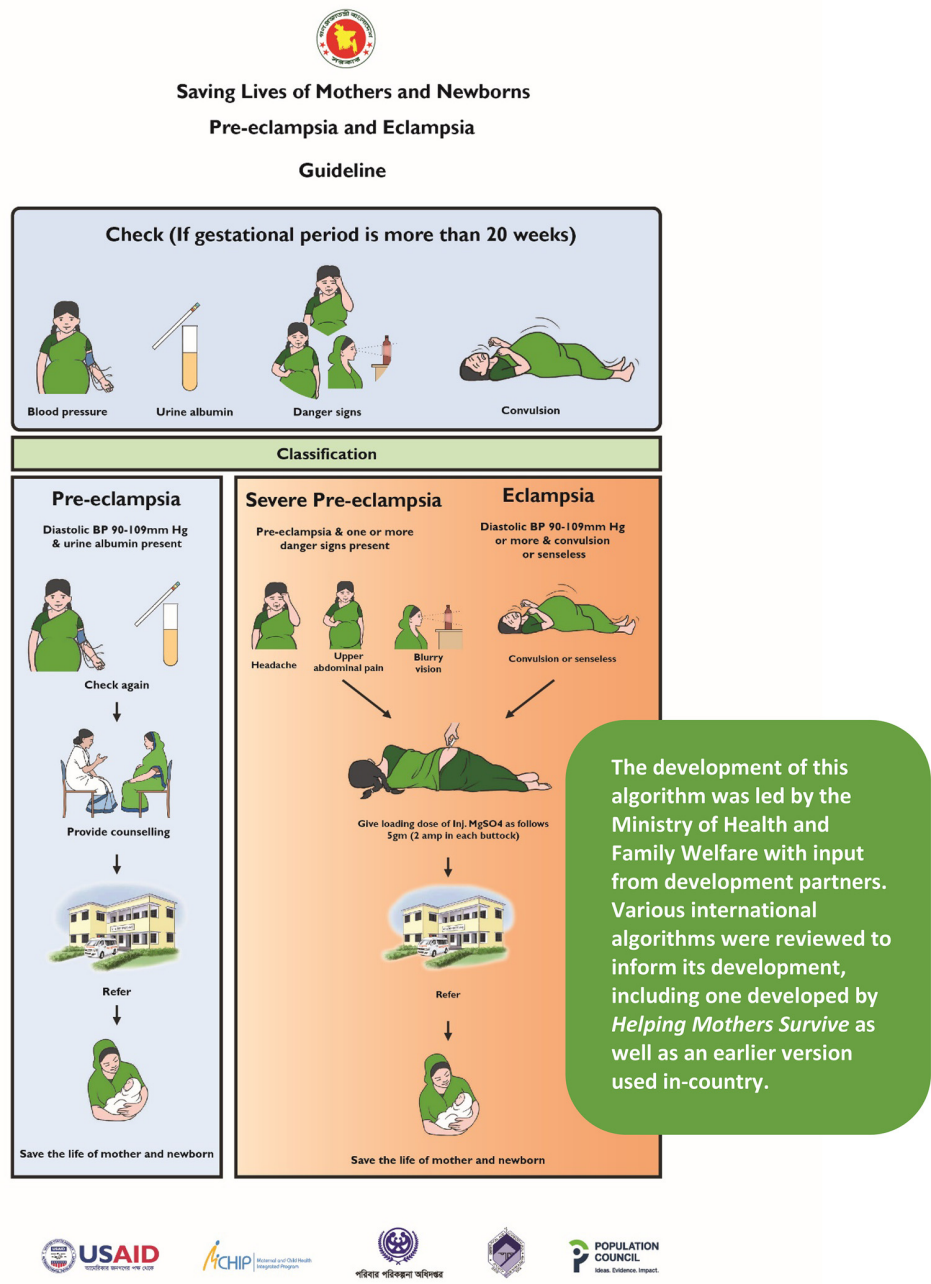
Pictorial Algorithm for the Management of Women With PE/SPE/E Developed by the Ministry of Health and Family Welfare, Bangladesh Abbreviations: BP, blood pressure; MgSO_4_, magnesium sulfate; PE/SPE/E, preeclampsia, severe preeclampsia, or eclampsia.

Following a pilot test conducted in 2013 and 2014, the National Technical Committee of the Directorate General of Family Planning (under MOHFW) endorsed a protocol for the identification and pre-referral management of severe preeclampsia and eclampsia at union-level facilities by the FWVs and SACMOs. The protocol recommended that all pregnant women receive at least 4 quality antenatal check-ups and that measurement of BP, urinalysis for proteinuria, and screening for the presence of SPE/E danger signs should be done at every antenatal, intrapartum, and postnatal service visit. All women identified with severe preeclampsia or eclampsia (see case definitions in Figure 2) were to be given a loading dose intramuscular injection of MgSO_4_ and then referred to the nearest comprehensive emergency obstetric and neonatal care (CEmONC) facility. For women with preeclampsia, the protocol specified that they should be referred to a nearby CEmONC facility for treatment with antihypertensive drugs and monitoring. Primary care providers in Bangladesh are not authorized to prescribe or administer antihypertensive drugs to pregnant women. A pictorial algorithm (Figure 3) was developed by MOHFW together with development partners as a job aid for frontline providers at UH&FWCs to guide them through triage and management of women with PE/SPE/E in line with the national protocol. Subsequently, MaMoni HSS selected 45 UH&FWCs for early implementation of this protocol as part of the larger set of maternal health interventions under the project.

This article presents findings from a record review of facility-level data on PE/SPE/E services at 35 of the 45 UH&FWCs, and it additionally provides recommendations for future efforts to address eclampsia at the community level within Bangladesh and in other low-resource settings.

We conducted a record review of facility-level data on PE/SPE/E services at 35 primary care facilities in Bangladesh.

## METHODS

### Facility Selection

**FIGURE 4:**
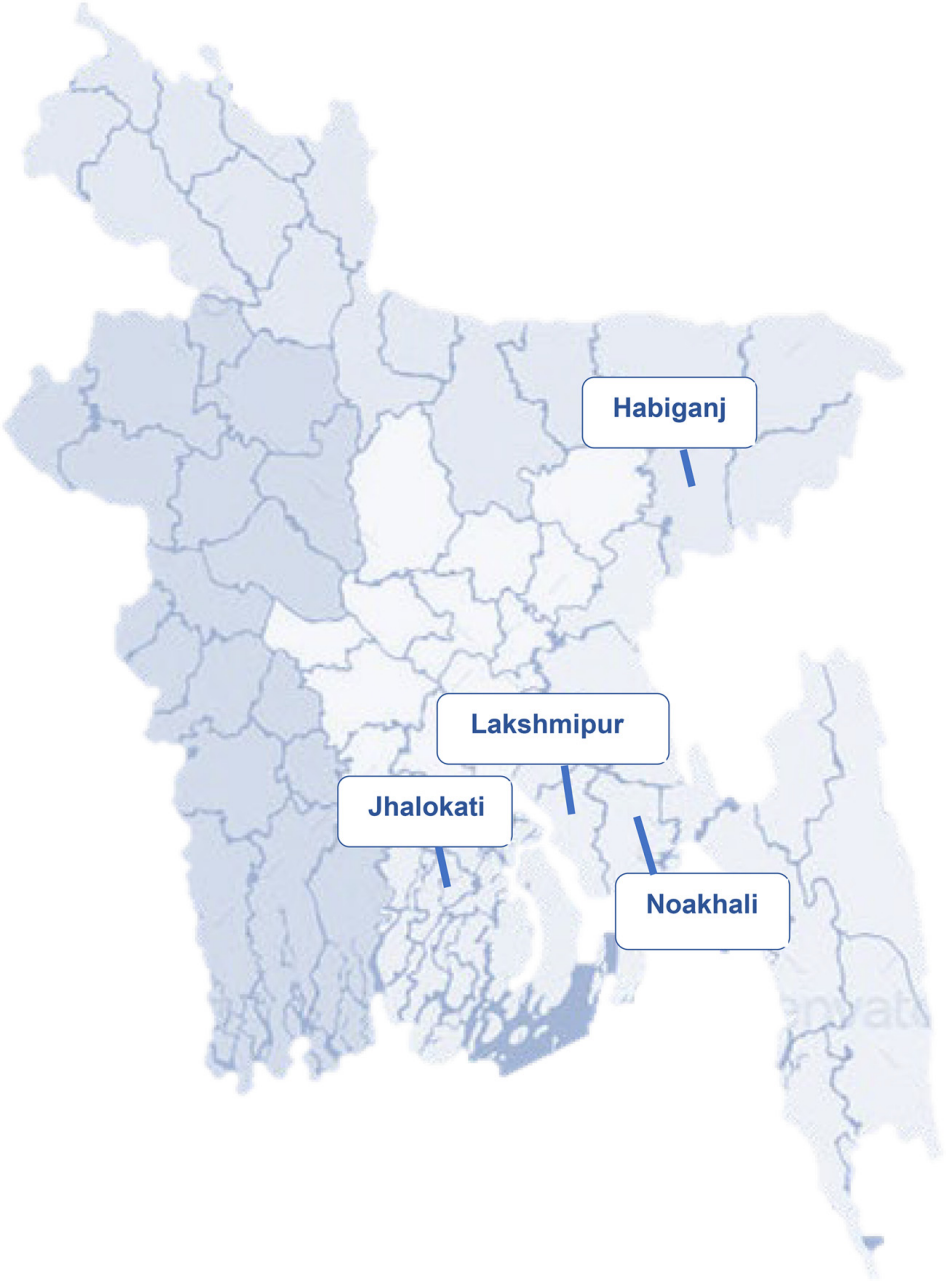
Map of Bangladesh Showing the Intervention Focus Districts

The 45 UH&FWCs initially selected to receive the PE/SPE/E intervention were chosen with consideration for having relatively high ANC coverage, having a resident FWV around the clock, having a referral facility at an accessible distance, and having outreach services with comparatively strong performance. All facilities were located in 4 districts (Figure 4) where the project focused on strengthening primary- and secondary-level public services to provide a complete package of evidence-based MNCH interventions, including family planning and nutrition.

### Initiation of the Intervention

To initiate the PE/SPE/E intervention, sensitization meetings were held with relevant district- and subdistrict-level health officials from the 4 districts together with representatives from MOHFW, members of the Obstetrical and Gynaecological Society of Bangladesh (OGSB), and MaMoni HSS program managers. A baseline survey carried out across all 45 facilities consisted of a retrospective record review of 6 months of facility-level data (covering the period October 2015 to March 2016) to identify documented cases of preeclampsia and eclampsia and how they were managed. The data source for the baseline was a new MNCH patient register that the project had earlier worked with MOHFW to distribute and train providers on how to use, as a replacement to using 4 separate registers to capture the same information. In addition, a routine service delivery point survey conducted quarterly by the project was used to check whether MgSO_4_ and BP apparatuses were present at each UH&FWC.

**FIGURE 5:**
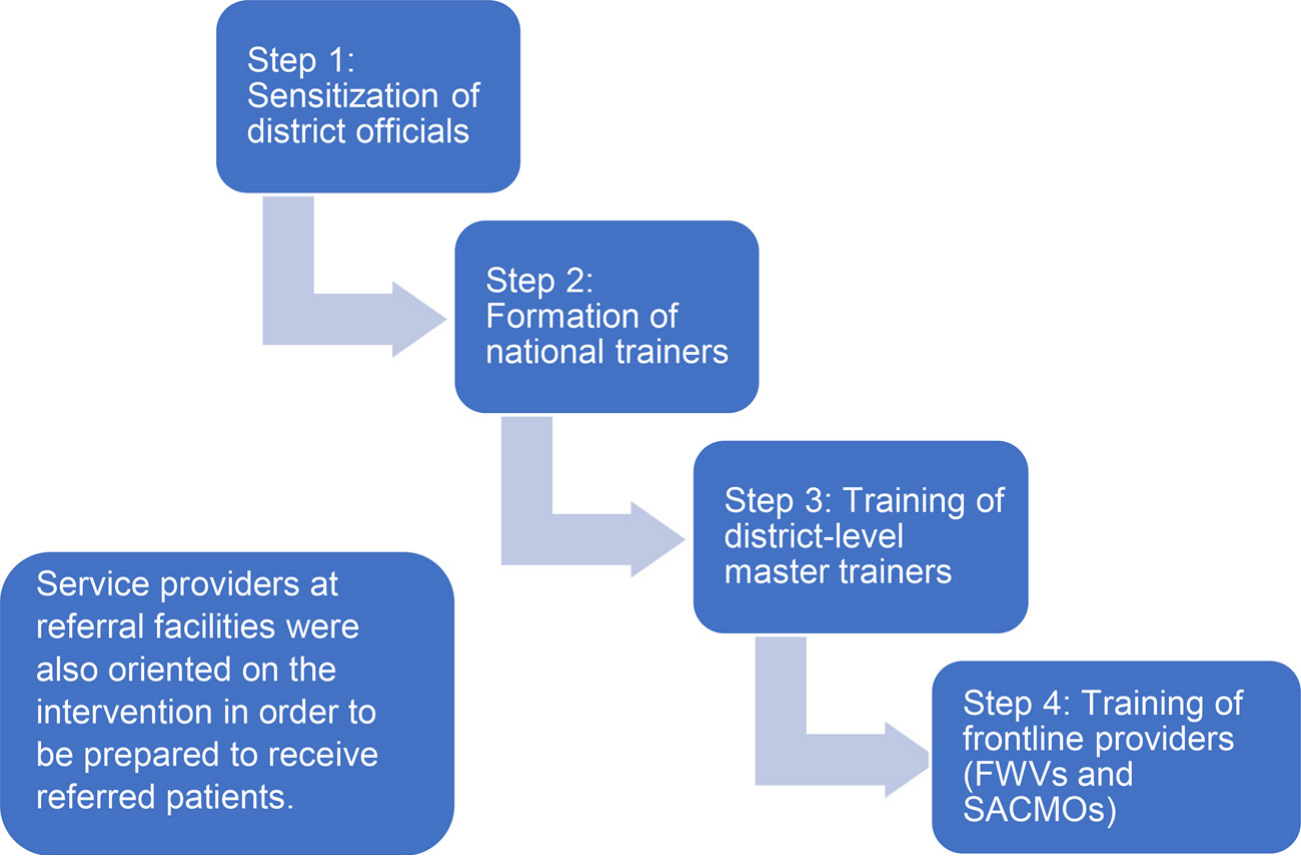
Cascade Training Model Abbreviations: FWV, family welfare visitor; SACMO, sub-assistant community medical officer.

A memorandum of understanding was developed between MaMoni HSS and OGSB in order to roll out training for FWVs and SACMOs in the targeted facilities. OGSB developed the training materials and carried out a cascade training approach in which national-level expert trainers established a group of district-level master trainers who then replicated the training at the community level for FWVs and SACMOs as new facilities adopted the intervention. Service providers at secondary-level referral facilities also received an orientation from OGSB on the intervention to prepare them to receive and manage the referred cases (Figure 5).

### Provider Training

Two-day competency-based trainings for frontline providers at the 45 facilities were carried out between March and May of 2016. In the trainings, providers were taught how to check BP using both digital (Microlife brand) and manual BP cuffs, conduct a urine protein analysis, and screen all women for danger signs. Training participants learned to identify PE/SPE/E based on the case identification criteria in Figure 2. They were taught how to administer a loading dose of MgSO_4_ via intramuscular injection and refer identified SPE/E cases to the nearest CEmONC facility. Each participant was provided with a digital BP machine and a laminated copy of the patient algorithm (that included both the pictorial version and a 1-page text description of the algorithm). Eclampsia kits that consisted of 4 preloaded vials of injectable MgSO_4_ were purchased by the project and allocated to UH&FWCs based on a rough estimate of possible eclampsia incidence. Incidence estimates were produced following a 2-step process. First, an initial calculation was made of the crude birth rate in each UH&FWC catchment area using data from the 2011 Bangladesh census. Then, calculations of PE and E incidence for each catchment area were made based on estimates of PE and E incidence (PE 2.8% of live births and eclampsia 2.3% of PE) in developing countries published by EngenderHealth in a 2007 report.^17^ Based on these projections, FWVs were provided with a monthly supply of MgSO_4_, which they restocked periodically from subdistrict drug storage facilities when conducting general inventory restocking as part of their regular work. Urine test tubes and strips for measuring albumin were already available at all primary care facilities through the existing supply chain.

Upon completion of the training, providers began screening for PE/SPE/E at their facilities and managing SPE/E with a pre-referral loading dose of MgSO_4_. Services were documented in the facility's MNCH register by FWVs in fields designed to capture key details of ANC, intrapartum, and PNC services. A single initial record was supposed to be created in the MNCH register for all women when they received ANC, intrapartum, and/or PNC services. At each new visit (e.g., second or third ANC visit, intrapartum care following ANC, or PNC following intrapartum care) information about that visit was to be added to women's initial record. A second record was to be created in the supplemental reporting form (hereafter referred to as a “patient linelist”) only for women diagnosed with SPE/E. The purpose of the patient linelist was to provide condensed essential SPE/E reporting information to MaMoni HSS. This form did not include variables related to ANC, intrapartum, or PNC services, nor did it record information about referrals for women with PE. However, it captured outcome information not recorded in the MNCH registers, such as whether referrals were completed and maternal and newborn outcomes.

### Complementary Program Inputs

The project's other maternal health interventions—to increase ANC coverage and quality, raise awareness at the community level about PE/SPE/E danger signs and the newly available services, improve the quality of CEmONC services at referral facilities, and monitor and improve the availability of essential drugs and supplies—complemented the efforts to improve PE/SPE/E identification and management at UH&FWCs. As part of its overall scope, the project also carried out monthly monitoring and supervision visits at selected facilities with district- and subdistrict-level managers. Facilities at different levels of the health system that were supported by the project's various MNCH interventions (Figure 1) received these supervision visits, including some of the 35 UH&FWCs included in this article. During these visits, a standard monitoring checklist was completed to facilitate review of a broad range of service quality issues, including around PE/SPE/E. Additional monitoring and supervision was carried out between January and July 2017 by OGSB together with MaMoni HSS managers and local-level health officials to specifically assess PE/SPE/E service provision at 8 UH&FWCs—1 high-performing and 1 low-performing UH&FWC in each focus district.

### Data Analysis

A secondary analysis of data from MNCH registers and patient linelists covering the period from September 2016 to August 2017 was carried out to develop a point estimate of correct initial management of PE/SPE/E identified during ANC, intrapartum, and PNC visits at 35 of the 45 intervention facilities. The 10 facilities not included in the analysis were no longer adequately staffed or had structural problems that prevented them from providing consistent ANC services during this time. Photocopies of MNCH registers were made by FWVs, transported to Dhaka in sealed boxes, and entered into an Excel spreadsheet for analysis. Patient linelists were already kept in password-protected electronic files in the MaMoni HSS Dhaka office. The analysis was designed to generate descriptive statistics summarizing key variables that reflect compliance with the PE/SPE/E screening and management protocol.

The variables analyzed included the proportion of women screened for PE/SPE/E, the proportion with indications of PE/SPE/E, and the percentage of those identified who received a loading dose of MgSO_4_ and referral to a higher level of care. Screening was determined by looking at relevant variables across ANC, intrapartum, and PNC visit records. Key variables in ANC records included diastolic blood pressure (dBP) and proteinuria, as well as open text fields for capturing pregnancy danger signs, patient “complaints and disease,” and provider “treatment and advice.” Intrapartum records included check boxes for blurred vision, severe headache, and convulsions, as well as a general “delivery complications” field and write-in fields for treatment and referral information. BP and urinalysis are required during intrapartum care, but these variables were not available in the intrapartum records. PNC records captured dBP and general write-in fields for complaints and disease and for treatment and advice, but not urinalysis. Write-in fields across all 3 of these services were closely examined and cleaned to establish uniformity of the presentation of key information. This clean-up primarily consisted of ensuring that all instances of treatment with magnesium sulfate were written as “MgSO_4_” and creating coded columns for PE/SPE/E cases and for referred cases. In addition to the generation of the point estimate, cases with a documented PE/SPE/E diagnosis were also compared with cases with only the indications of PE/SPE/E documented but not the actual diagnosis. The final point estimate merged the findings from the analysis of both the MNCH registers and the patient linelists.

Ethical approval for this analysis was granted from the Johns Hopkins School of Public Health Institutional Review Board as well as the Bangladesh Medical Review Council's National Research Ethics Committee.

Other analyses were also carried out to assess facility readiness and provider competency. Results from a quarterly service delivery point survey managed by MaMoni HSS were reviewed to verify the presence of MgSO_4_ and BP machines at each of the 35 UH&FWCs covering the periods January–March 2016 and July–September 2017, as well as just prior to and at the end of the period of analysis. A short questionnaire was completed by field-level MaMoni HSS staff in April 2018 to check for the presence of the laminated algorithm, test tubes, and urinalysis strips at each UH&FWC. This questionnaire also double-checked for the presence of MgSO_4_ and BP machines. Both of these datasets were used to ascertain facility readiness. Provider knowledge was assessed by analyzing results from pre- and post-training questionnaires with 32 items that checked providers' knowledge of the PE/SPE/E competencies covered in the training. Qualitative analysis consisted of reviewing the reports from the OGSB-led supervision visits, as well as reports from the project's joint supervision visits and quarterly reports that had been submitted to USAID to gather contextual information to inform the program description and discussion.

## RESULTS

Results from the baseline revealed that providers had documented 3 cases of PE and 2 cases of eclampsia between October 2015 and March 2016. None of the women with documented eclampsia were treated with MgSO_4_. Analysis of the service delivery point dataset revealed that prior to the intervention, MgSO_4_ was not present at any of the UH&FWCs. All but 4 had BP machines. These machines were assumed to be manual BP gauges, which are provided to facilities through the national supply chain, although the type of machine was not indicated in this dataset. In the second service delivery point survey covering July–September 2017, all 35 facilities had MgSO_4_ and BP machines. The questionnaire completed in April 2018 showed that all 35 facilities had the laminated PE/SPE/E algorithm and urinalysis test tubes and strips. At that time, 33 of the 35 facilities reported having a BP machine. The 2 that did not have a BP machine noted that the FWVs were using their own personal BP machines in the facilities. An additional 3 facilities (which reported having BP machines) noted that they were using manual machines that were not giving correct readings. Two facilities reported stock-outs of MgSO_4_ at the time they completed the questionnaire (Table 1). On average, providers correctly answered 18 out of 32 questions (or 57%) on the pretest and 26.25 out of 32 (82%) on the posttest.

**TABLE 1.** Facility Readiness Survey Results, July–September 2017

Readiness Indicators	Availability in the Facilities
PE/SPE/E pictorial algorithm	Present at all 35 facilities
Test tube for albumin test	Present at all 35 facilities
Urine strip for albumin test	Present at all 35 facilities
BP machine	2 facilities did not have a BP machine; FWVs used their personal BP machines instead. 3 facilities reported having a BP machine that gave incorrect readings.
MgSO_4_	Missing at 2 facilities

Abbreviations: BP, blood pressure; FWV, family welfare visitor; MgSO_4_, magnesium sulfate. PE/SPE/E, preeclampsia, severe preeclampsia, or eclampsia.

The following summary of the facility-level data comprises analyses of both the MNHC register data with ANC, intrapartum, and PNC service records and the patient linelists with only women who had an SPE/E diagnosis. Missing records appeared to be common in both datasets. For example, 25 women who had SPE/E documented by an FWV in the MNCH register did not appear in the patient linelist. Likewise, 88 women with SPE/E were reported to MaMoni HSS via the patient linelist, but their records did not appear in the MNCH register.

Across both datasets, 13,346 women were seen for ANC, intrapartum, and/or PNC services at the 35 UH&FWCs between September 2016 and August 2017. The MNCH registers contained records of 13,031 ANC visits, 3,641 intrapartum visits, and 5,833 PNC visits. The patient linelists contained records of 139 women with SPE/E whose diagnoses were reported to MaMoni HSS. Records from only 51 of these women also appeared in the MNCH register.

Analysis of the MNCH registers revealed that most pregnant women (9,898, 74%) were between 20 and 29 years of age (Table 2). A total of 8,462 (65%) pregnant women received just 1 ANC consultation during pregnancy, while 2,358 (18%) received 3 or more (Table 3).

**TABLE 2.** Age Distribution of Women Who Received ANC, Delivery Services, and/or PNC (N=13,346)

Mother's Age	No. (%)
<20	1,728 (13)
20–24	5,802 (43)
25–29	4,096 (31)
≥30	1,564 (12)
Not recorded	156 (1)

Abbreviations: ANC, antenatal care; PNC, postnatal care.

Source: Maternal, newborn, and child health register.

**TABLE 3.** Distribution of Pregnant Women by Total Number of ANC Consultations Received (N=13,031)

ANC Visits	No. (%)
1	8,462 (65)
2	2,211 (17)
3	1,787 (14)
≥4	571 (4)

Abbreviation: ANC, antenatal care.

Source: Maternal, newborn, and child health register.

**FIGURE 6:**
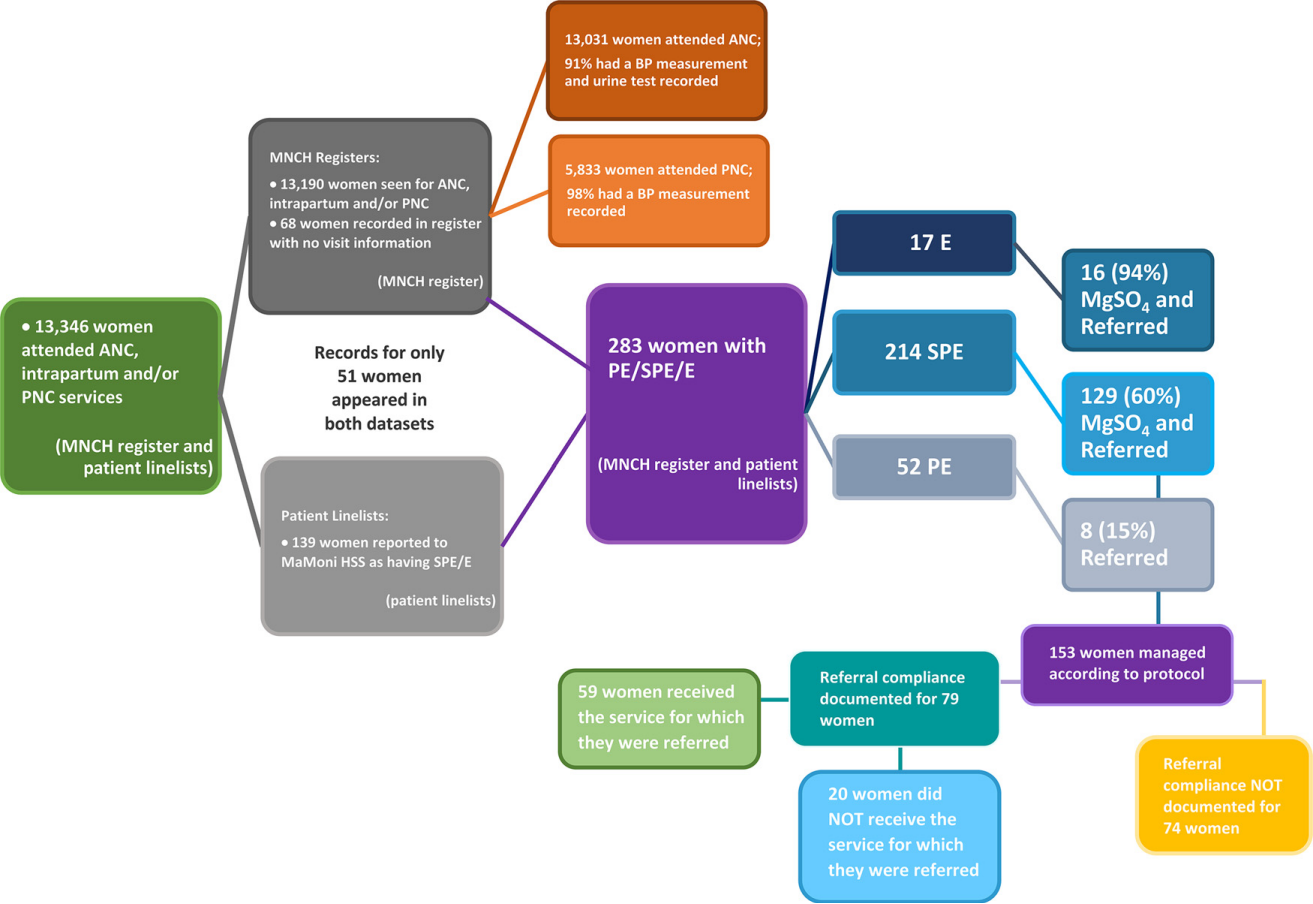
Client Flow Chart Abbreviation: ANC, antenatal care; BP, blood pressure; HSS, Health Systems Strengthening; MgSO_4_, magnesium sulfate; MNCH, maternal, newborn, and child health; PE/SPE/E, preeclampsia, severe preeclampsia, or eclampsia; PNC, postnatal care.

Both a dBP and a proteinuria reading were recorded at over 90% of ANC visits. Among the 5,833 PNC visits documented in the MNCH register, dBP was recorded 98% of the time. Across both datasets, 283 women were identified as having PE/SPE/E—52 preeclampsia, 214 severe preeclampsia, and 17 eclampsia (Figure 6). An additional 250 women without PE/SPE/E had documented hypertension (dBP≥90). In the MNCH register, 53% of PE/SPE/E cases had been identified by a provider, meaning that a written diagnosis of PE/SPE/E or treatment with MgSO_4_ was recorded. The rates at which the clinical management of identified cases followed the national protocol are depicted in Figure 6. Overall, the records indicated that providers adhered to the protocol for 54% of women with PE/SPE/E (153 women). Adherence to the protocol was lowest for PE—only 15% of women with PE were referred. Adherence was highest for eclampsia, with a loading dose of MgSO_4_ being administered and a referral being made to a CEmONC facility for 94% of women with eclampsia.

Among the PE/SPE/E cases that were not referred, 74 women (62%) were admitted to a UH&FWC for delivery. Among women with SPE/E who were referred, referral compliance was only documented for about half (79, 52%). Of this group, 59 (75%) complied with their referral, while 20 (25%) did not.

Among the women who were referred, type of delivery was recorded for 147. Of these, 118 (80%) had a vaginal birth, while 29 (20%) had a cesarean delivery. Newborn outcomes were recorded for 154 (54%) of all PE/SPE/E cases. Among those with recorded newborn outcome information, 150 (97%) of deliveries resulted in a live birth. There were 3 stillbirths and 1 newborn death. In all 4 of these cases, the mother had been referred to a higher of level of care.

## DISCUSSION

We aimed to generate a point estimate of correct diagnosis and initial management of PE/SPE/E by frontline providers at primary care facilities in Bangladesh. We found that frontline providers managed slightly over half of women with PE/SPE/E in line with their training. Significant challenges were noted with regard to the quality of the data. This issue stood out in the analysis because large numbers of women with SPE/E were present in the MNCH register but missing from the patient linelists, and vice versa. The range of missing information in the MNCH register, as well as the disparity between women whose diagnosis was documented by a provider versus identified in the analysis based only on dBP, proteinuria, and/or a danger sign, further highlighted the data quality challenges.

We found that frontline providers managed slightly over half of women with PE/SPE/E in line with their training.

Two possible explanations could account for the lack of record duplication expected between the MNCH register and the patient linelists. First, frontline providers may have sometimes established a record for a woman in the MNCH register but not the linelist (and vice versa). Second, some of the relevant MNCH registers may not have been shared from all 35 facilities. Both of these scenarios could potentially explain why 88 women with SPE/E were reported to MaMoni HSS in the patient linelists but not found in the MNCH register.

Another quandary was that the prevalence of PE was much lower than that of both hypertension and SPE. Typically, a declining pattern would be present, with the highest numbers of women having hypertension, fewer women having PE, and many fewer women having SPE/E. This pattern not being apparent in our dataset is likely explained by inaccurate measuring or recording of BP and/or proteinuria (in addition to the issues with missing data already described). BP measurement and/or urinalysis may not have been done at all (despite a reading having been recorded) or may have been done incorrectly. Further, BP measurements may have been rounded up or down when recorded. Rounding up could have skewed the results toward higher numbers of SPE cases than there actually were. For women with a dBP measurement of ≥90, if a negative proteinuria measurement was recorded but the test was not actually done, records may have fallen into the category of hypertension, rather than PE. Anecdotal evidence from both project staff and local researchers studying FWV skills in detecting and managing PE/SPE/E supports that any of these may be realistic scenarios. While not documented thoroughly enough to be fully substantiated, these scenarios are also supported by some published literature.^3^^,^^18^

The data quality challenges highlighted the need for ongoing mentoring, support, and refresher training for frontline workers. This need is also apparent in the low rate of adherence to the PE/SPE/E protocol and is emphasized in the broader literature on this topic as well.^3^^,^^5^^,^^8^^,^^10^ Furthermore, while FWVs' adherence to the standard protocol requires more intensive monitoring and guidance, improvements in recordkeeping are also essential for accurate tracking of service quality and case management. In an ideal scenario, a rapid feedback loop would exist in which service data would be regularly consolidated and summarized, and trends and issues discussed and addressed directly with FWVs and SACMOs.

Other challenges are the availability of MgSO_4_ and functioning BP machines. MgSO_4_ being on the government's essential drug list is an advantage, but if MOHFW cannot fund its availability at the primary care level, then its supply is dependent on donor funds and is not sustainable. Finally, more attention should be paid to ensuring that frontline providers have access to functioning BP apparatuses and use them correctly. The project's scope was limited to providing replacement devices to facilities where providers reported problems with the functionality of the digital BP machine they had been provided with. However, maintaining proper calibration of aneroid devices and ensuring correct measurement techniques are fundamental challenges, particularly in low-resource settings.

## CONCLUSION

Community-based management of PE/SPE/E is an important maternal health intervention that is being tested in a variety of community-level and primary care settings in low- and middle-income countries. Findings from program experience in Bangladesh indicate that intensive inputs are required to introduce and maintain quality of PE/SPE/E service delivery in primary care facilities. The findings also demonstrate that delivering competency-based training together with the provision of essential supplies (i.e., BP machines, a visual job aid, and injectable MgSO_4_), supportive supervision, and complementary program inputs at the national, community, and secondary care levels are effective interventions to begin to enable frontline providers to comply with PE/SPE/E screening and management protocols. In addition to these inputs, well-functioning BP apparatuses, routine monitoring of facility-level data, and ongoing performance management are also critical for providing and monitoring quality services. These findings can contribute to strengthening community-level PE/SPE/E interventions in Bangladesh and in other low-resource primary care settings.

## Notes

**Peer Reviewed**

**First Published Online:** September 16, 2019
